# A case of co-existing paraganglioma and thymoma

**DOI:** 10.1186/s40064-015-1269-z

**Published:** 2015-10-21

**Authors:** G. Bano, D. Sennik, M. Kenchaiah, Ye Kyaw, Katie Snape, V. Tripathi, P. Wilson, I. Vlahos, I. Hunt, S. Hodgson

**Affiliations:** Department of Endocrinology and Diabetes, Thomas Addison Unit, St George’s Healthcare NHS Trust, Blackshaw Road, Tooting, London, SW17 0QT UK; Clinical Genetics, Southwest Thames Regional Genetics Service, St George’s Healthcare NHS Trust, London, UK; Cellular Pathology, St George’s Healthcare NHS Trust, London, UK; Radiology, St George’s Healthcare NHS Trust, London, UK; Cardiothoracic Surgery, St George’s Healthcare NHS Trust, London, UK

**Keywords:** Hereditary paragangliomas, Thymoma, Carotid body tumour, Mediastinal mass, Succinate dehydrogenase (SDH) subunits

## Abstract

**Background:**

Head and neck paragangliomas are rare tumours and can arise as a part of inherited syndromes. Their association with thymic tumour is not well known.

**Case description:**

This report describes a female patient who presented with right sided neck paragangliomas. The histology of the tumour was consistent with paraganlioma. Few years later her MRI scan of the chest revealed presence of an anterior mediastinal mass that corresponded to the location of the thymus. Review of her previous scans showed that the mass was present all along and had gradually increased in size. Patient developed symptoms including fatigue, dyspnoea, migratory polyarthritis, Raynaud’s phenomenon and erythema nodosum. She had sternotomy and excision of mediastinal mass. The histology was consistent with cortical thymoma (WHO type B2) and she had radiotherapy. After treatment her constitutional symptoms improved. Her paraganglioma susceptibility genes are negative.

**Discussion and evaluation:**

To our knowledge this is only the second case report in the literature of coexistence of carotid body tumour and thymoma. The first case reported was bilateral carotid body tumour, thyroid gland adenoma and thymoma. This case also highlights the importance of long term surveillance, multidisciplinary management and being aware of associated pathologies in patients with isolated paraganglioma.

## Background

Head and neck paragangliomas (HNPGLs) are tumors of the autonomic system. They arise from specialised neural crest chromaffin cells of the parasympathetic paraganglia of the skull base and neck, and are also called glomus tumors. HNPGLs account for approximately 3 % of all paragangliomas (PGLs). Most often, HNPGLs progress slowly are benign and nonsecreting with some carotid body tumors being reported to exist for many years as a painless lateral mass on the neck. They can be widely distributed and prominent locations are the carotid body tumour (CBT) along with the vagal, jugular, and tympanic glomus. Symptoms depend on the specific locations (Taïeb et al. 2014; Boedeker et al. 2005). HNPGLs have been identified with many susceptibility genes: NF1, RET, VHL, SDHA, SDHB, SDHC, SDHD, SDHAF2 (SDH5), IDH1 and TMEM127. Hereditary HNPGLs are mostly caused by mutations of the SDHD gene, but SDHB and SDHC mutations are not uncommon in such patients. Multiple head and neck paragangliomas are common in patients with SDHD mutations, while malignant head and neck paraganglioma is mostly seen in patients with SDHB mutations (Burnichon et al. 2010; Neumann et al. 2011; Schiavi et al. 2005).

The treatment of choice is surgical resection but this can be challenging because of the tumors’ location in the vicinity of important blood vessels and cranial nerve.

We report a 49 year old female who presented with a right sided neck mass and earache. After standard diagnostic procedures and surgical removal the diagnosis of paraganlioma was confirmed. She was found to have an anterior mediastinal mass on a screening MRI and 5 years later this turned out to be a cortical thymoma (WHO type B2). We also present a brief review the literature regarding the coexisting neck and mediastinal masses. To our knowledge this appears to be only the second case of paraganglioma associated with thymoma described in the literature.

## Subjects and methods

### Case history

**Fig. 1:**
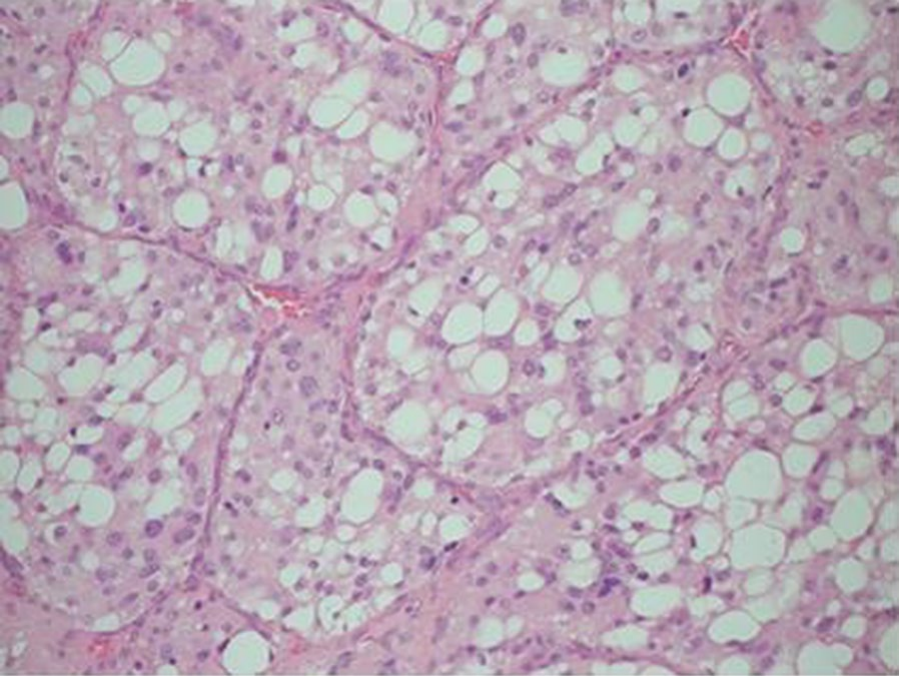
HE stain of paraganglioma

**Fig. 2:**
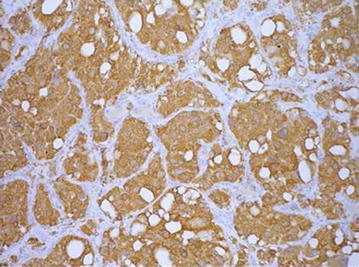
Immunohistochemistry of glomus vagale tumour showing NSE stating in neuroendocrine cells

The patient is a 49 year old lady who initially presented with right sided earache and right sided neck swelling. Following ear, nose and throat (ENT) assessment, surgical excision of the mass together with an adjacent lymph node was performed. At operation the mass was consistent with a glomus vagale tumour. The histology revealed organised growth pattern in which tumour cells formed characteristic nests (Zellballen pattern) separated by fibrovascular connective tissue septa. The tumour cells were polygonal with large nuclei, many with prominent nucleoli and plentiful eosinophilic granular cytoplasm. The tumour stained positively for synaptophysin, NSE and chromogranin A. No obvious metastatic features were found. The lymph node histology revealed reactive changes. The histology was consistent with paraganglioma (Figs. 1, 2). Post-operatively she suffered the complications of vagal nerve palsy and pulmonary embolism.

**Fig. 3:**
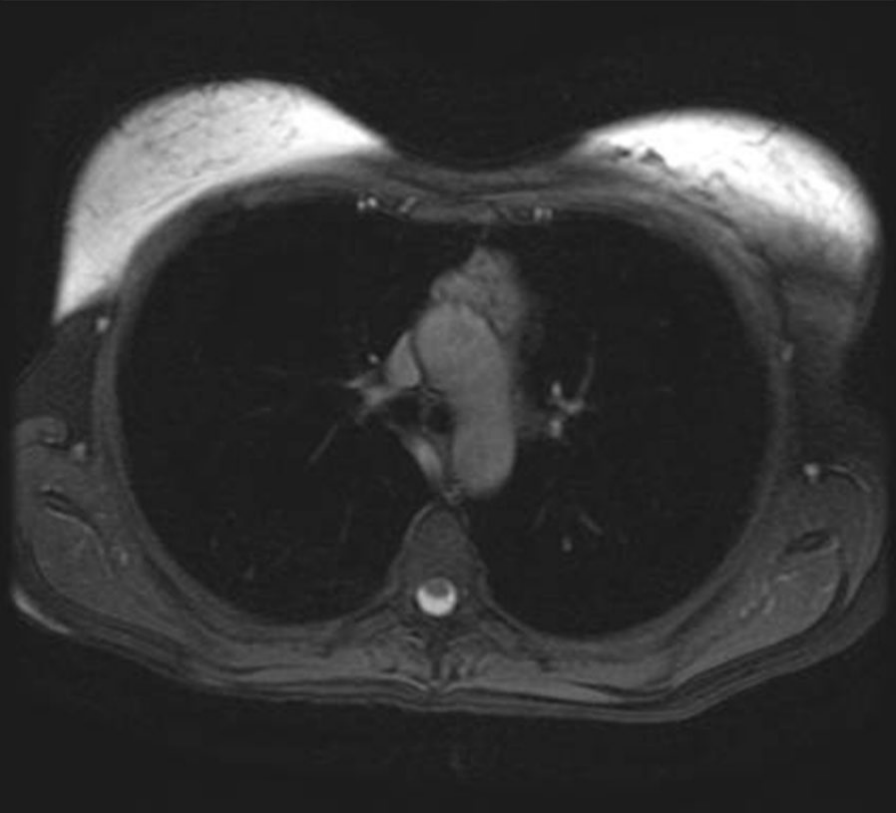
Anterior mediastinal mass

**Fig. 4:**
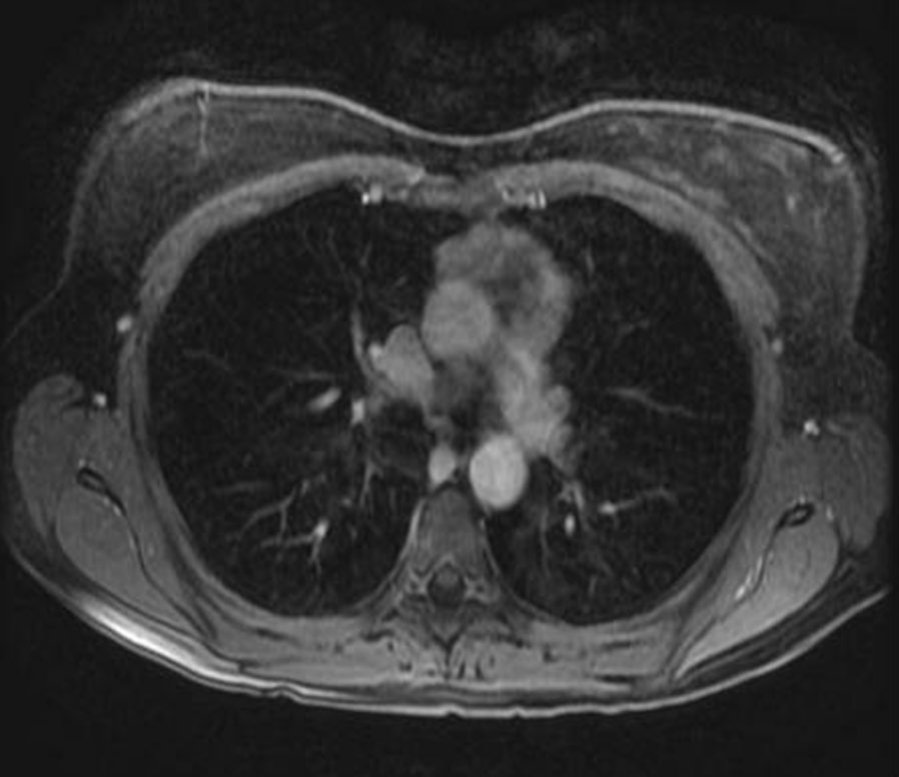
Increase in the size of mediastinal mass

Five years later she was seen in Genetic Endocrine clinic and had an ultrasound scan of the neck, MRI scan of the abdomen and thorax as a part of screening for paraganglioma follow up. MRI scan revealed the presence of a 4 × 2 cm anterior mediastinal mass (Fig. 3). This mass corresponded to the location of the thymus and had central calcification. Review of her previous CT pulmonary angiogram (CTPA) done following her neck surgery 5 years earlier revealed that the mass was eve present on this scan. This mass had only marginally increased in size during this time (Fig. 4). It was decided to manage it conservatively and follow up with serial MRI scans. She had normal concentrations of urinary fractionated metanephrines.

**Fig. 5:**
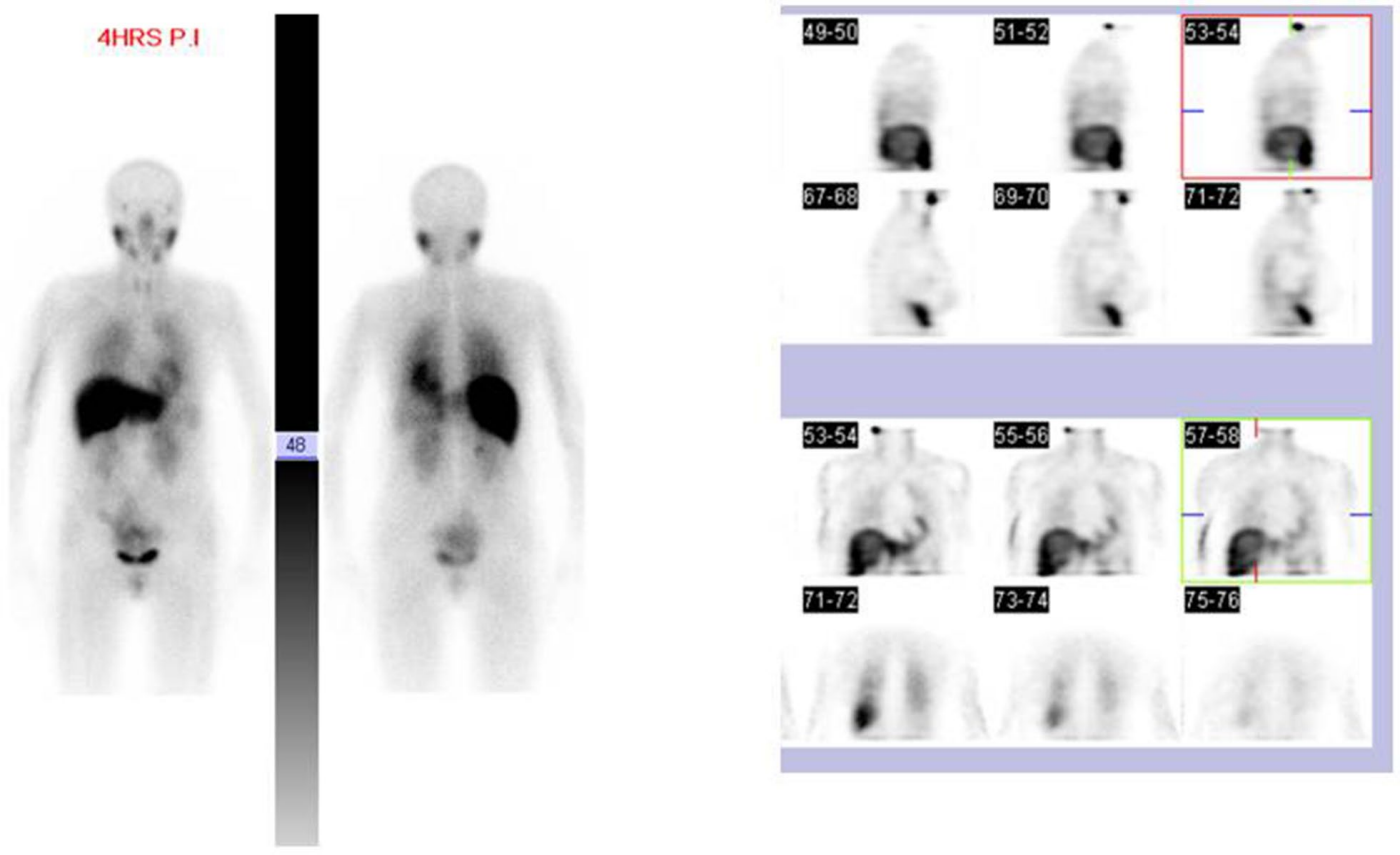
MiBG scan: no focal uptake in mediastinum

Two years later her annual scan revealed an increase in the size of mediastinal mass to 5.8 by 3.1 cm (Fig. 4) Whole body metaiodobenzylguanidine (MIBG) scintigraphy showed no uptake in the mass (Fig. 5). At this stage patient had noticed some additional symptoms including fatigue, hoarse voice and dyspnoea. She also described migratory polyarthritis of the small joints, Raynaud’s phenomenon and erythema nodosum. She was referred to the neurology team to exclude myasthenia gravis in view of her mediastinal mass. Acetylcholine receptor antibodies were negative.

**Fig. 6:**
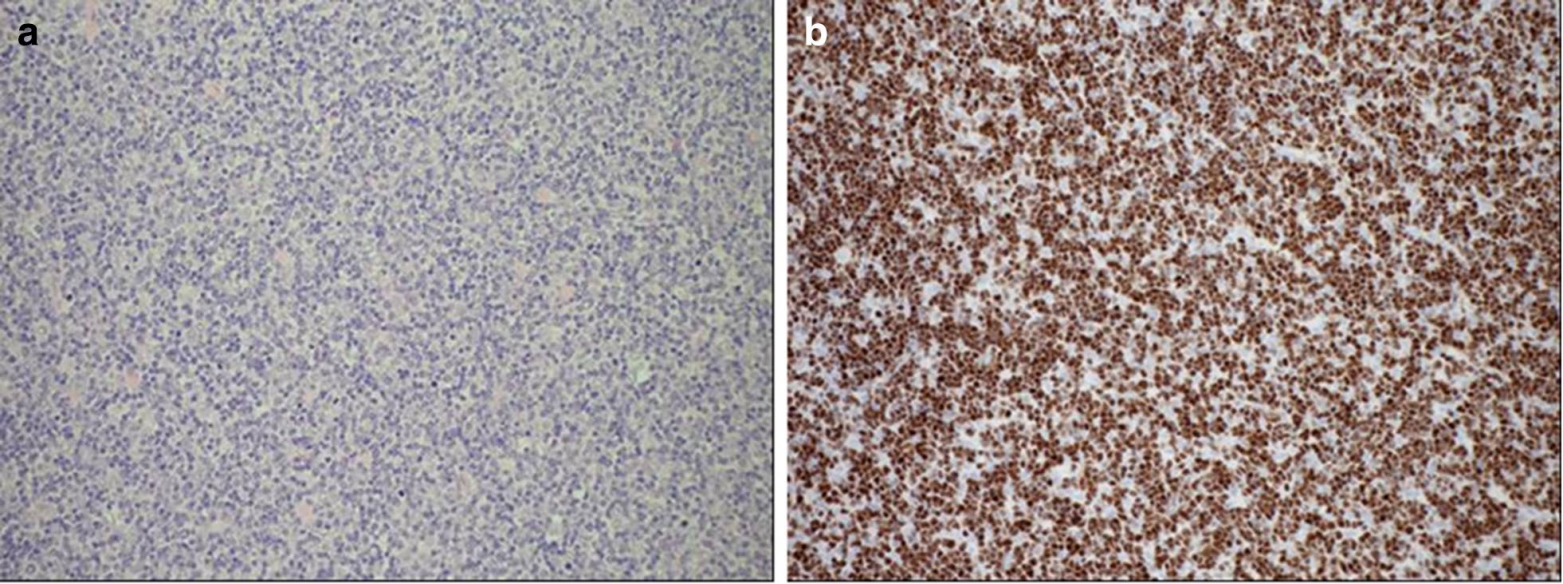
**a** The histology showed HE (hematoxylin and eosinophil) stained intermediate sized lymphocytes with cytoplamic processes. **b** Immunochemistry showing Tdt, CD1 and cd5 reactive T lymphocytes

In view of the enlargement of the mass and constitutional symptoms patient was discussed in a multidisciplinary meeting and the decision was made to operate. She underwent median sternotomy and excision of mediastinal mass. The mass was completely excised. The histology showed intermediate-sized lymphocytes with admixed epithelial cells with cytoplasmic processes. Immunohistochemistry revealed the presence of reactive T lymphocytes; TdT, CD1a and CD5 positive (Fig. 6). The histology was consistent with cortical thymoma (WHO type B2). She made a good postoperative recovery and was referred for radiotherapy. Patient is currently very well with no symptoms and 1 year after operation, imaging investigations are still normal.

### Genetic testing

Patient signed a written informed consent for the test. Screening for germline mutations of the *RET*, *VHL*, *SDHB*, *SDHC*, *SDHD*, *SDHAF2* and *TMEM127* was negative. Analysis of the Paraganglioma/Phaeochromocytoma gene panel was undertaken in the West Midlands Regional Genetics Laboratory. The panel uses an Illumina MiSeq platform to capture the coding regions of the SDHAF2, SDHB, SDHC, SDHD, RET (exons 10, 11, 13–16), MAX, TMEM127 and VHL genes using the TruSight Cancer Panel target enrichment system (v1, Illumina). Sanger sequencing is used to confirm any variants identified. MLPA analysis of all exons of the VHL gene (MRC-Holland Kit: P016-C2) and SDHB/C/D (MRC-Holland Kit: P226-C1) is undertaken.

## Discussion

HPGLs overall are rare tumours and are known to have hereditary-familial tendency. Their association with thymic tumour is not well known. To our knowledge thymoma associated with paraganglioma has been reported only once before in the literature (Refior and Mees 2000) and we report this second case of HPGL coexisting with a thymic tumor in an adult patient. Her mediastinal tumour was initially growing slowly and she was asymptomatic. 5 years after her initial surgery for paraganglioma she developed symptoms suggestive of compression of anterior mediastinal structures, autoimmune pathology and her imaging showed increase in the size of her mediastinal tumour. The tumor was presumed to be benign as there was no metastasis (i.e., lymph node or distant metastases) after imaging. Our patient had extensive biochemical testing. The mediastinal mass was not MIBG avid and her urinary metanephrines were normal. Her genetic testing for hereditary paragangliomas has been negative so far. For the coexistence of HPGLS and such tumors a common neuroectodermal origin has been proposed as an explanation. The hypothesis is supported by combined (mixed) thymoma-neuroendocrine tumours and the occurrence of either thymomas or thymic neuroendocrine tumours in MEN1 syndrome patients (Rashid and Cassano 2013).

Thymus has an important role in the development of an effective immune system as well as endocrine function. Thymus has two main components; the lymphoid thymocytes and the thymic epithelial cells. The thymic epithelium develops first from the third pharyngeal pouch as two flask-shaped endodermal diverticula and extend laterally and backward into the surrounding mesoderm and neural crest-derived mesenchyme. The mature thymus epithelium has two main cell types: cortical thymic epithelial (cTECs) and medullary thymic epithelial cells (mTECs) or stromal cells. These thymic stromal cells provide signals for T cell differentiation. During the late stages of the development of the thymic epithelium, hematopoietic bone marrow precursors migrate into the thymus. After this stage the normal thymic development is dependent on the interaction between the thymic epithelium and the hematopoietic thymocytes (Farley et al. 2013). Tumours of the thymus are extremely rare and comprise <1 % of all adult cancers. Thymoma is a benign tumour but has a malignant potential. There are two major types of thymoma depending on the neoplastic epithelial cell type. In type A thymoma the cells and their nuclei have a spindle or oval shape, and are uniformly bland. In type B thymoma the cells have a predominantly round or polygonal appearance. In 50 % of cases thymoma is detected as an incidental finding on imaging. It constitutes about 20 % of the mediastinal tumours so the differential diagnosis includes paraganglioma. In 95 % of cases it presents as an anterior mediastinal mass. Thymic tumours occur at almost all ages (range 7–89 years) with a peak incidence between 55 and 65 years. There is no pronounced sex predilection. Patients exhibit an increased incidence of second cancers irrespective of the histology of the thymic epithelial tumour. The etiology of thymic tumours is largely unknown. Some epidemiologic clustering of thymomas and neuroendocrine tumours has been observed among patients with multiple endocrine neoplasia (MEN1) syndrome. Epstein–Barr virus (EBV) infection may play a role in a minority of thymic carcinoma. Symptoms can be due to local complications such as superior vena cava syndrome, pleural or pericardial effusions or patients may have systemic symptoms such as fever or weight loss (Mikhail et al. 2012). In addition, thymomas can cause parathymic syndrome in 40 % of cases. These syndromes are often typical for a specific tumour type and may precede or follow thymoma resection. Thymomas can exhibit a spectrum of autoimmune phenomena, comprising neuromuscular, haematopoietic, dermatologic, rheumatic/vasculitic, hepatic and renal diseases. These are more commonly seen in type A and B thymomas as in our patient. Myasthenia gravis is more frequently associated with type B thymomas, while hypogammaglobulinaemia (Good syndrome) and pure red cell aplasia are more typical for type A thymoma. 20 % of patients can have non thymic cancers. Thymic carcinomas can occasionally be associated with syndrome of inappropriate secretion of antidiuretic hormone (SIADH). Carcinoid a neuroendocrine tumour of the thymus is well reported. One-third of these patients have Cushings syndrome due to ectopic ACTH production. 15 % of carcinoids can be associated with multiple endocrine neoplasia (MEN) syndromes mostly with MEN type 1 and some with MEN type 2. Thymic carcinoid tumours associated with MEN syndromes are mostly malignant and can present with bony metastasis (Kaltsas 2010).

Recurrent genetic alterations have so far been reported for thymomas as well as for thymic squamous cell carcinoma. Deletions of chromosome 6p are reported with type A thymoma and gains of chromosome 1q and losses of chromosomes 6 and 13q are reported with type B3 thymomas (Zettl et al. 2000).

Paragangliomas (PGLs) are found mostly in the neck and abdomen, less commonly in the pelvic sympathetic plexus of the urinary bladder, and rarely in the mediastinum. PGLs may occur sporadically or as part of a hereditary syndrome. 60 % can develop metastatic disease, indicating that these tumors are often aggressive and need follow up (Ghayee et al. 2009). Only 2 % of PGLs are found in the mediastinum and are associated with germ line mutations in either SDHB or SDHD. 30 % are associated with elevated catecholamines or metanephrines. Multiple endocrine neoplasia type 2, neurofibromatosis type 1, and von Hippel–Lindau syndrome are familial syndromes that predominantly predispose to adrenal pheochromocytomas. In our patient metanephrines were normal. Mutations in the genes encoding succinate dehydrogenase (SDH) subunits B, C, and D cause extra-adrenal PGLs. Other recently identified genes associated with PGLs are SDHA and TMEM127 (Offergeld et al. 2012).

The common neuroectodermal origin is thought to be the reason for coexistence of carotid body tumour and a thymic tumour as in our patient. This was proposed in the first case who presented with bilateral carotid body tumor, thyroid adenoma and a thymoma (Refior and Mees 2000). Neuroectodermal tumours are part of a family of tumours characterized by genotypic, immunophenotypic, and functional properties of neuroendocrine differentiation. Within thymus such tumors comprise lesions derived from neuroendocrine elements within the thymus, from paraganglionic rests, or from misplaced embryonal structures within the mediastinum. The most common neuroendocrine neoplasm of this anatomic region is the neuroendocrine carcinomas of the thymus which is relatively rare (Moran and Suster 2000). Such tumors can often also be the source of hormone secretion, either because of adrenocorticotrophic hormone (ACTH) secretion by the thymic carcinoid itself or its association with other endocrine neoplasms. These tumors manifest in one of four ways: (1) they may be asymptomatic, found incidentally on routine chest radiography, (2) they may produce symptoms of thoracic structure displacement or compression, (3) they may present with symptoms related to an associated endocrinopathy, or (4) they may present with symptoms and signs relating to a distant metastasis, most commonly to the liver, lung, pancreas, pleura, and bone. It has been estimated that over one-third of patients are asymptomatic and are incidentally discovered (Duh et al. 1987). More rarely, the mediastinum also can be the seat of tumors derived from aorticopulmonary or aorticosympathetic paraganglia or from ectopic or supernumerary parathyroid glands (Suster and Moran 2001).

The treatment options for HNPGLs comprise surgical resection, as well as irradiation therapy, stereotactic radiosurgery and permanent embolization. If necessary combined treatment strategy could be used. For carotid body tumours the principal is complete tumor resection. With complete surgical resection, the tumor is controlled locally in 89–100 % of cases. However, there is a possibility of postoperative cranial nerve dysfunction even in cases of successful surgical removal of CBT and complication rates are directly related to tumor size. Postoperatively our patient had vagal nerve palsy and patient is left with hoarse voice (Boedeker et al. 2005).

Complete surgical excision is the treatment of choice for non metastatic thymoma and thymic carcinoma, even when the tumor is locally advanced. This is followed by postoperative radiotherapy to decrease the incidence of local recurrence. In an advanced disease surgical debulking, radiotherapy, and chemotherapy are recommended (Berman et al. 2011). Our patient had surgical resection followed by radiotherapy and till date remains symptoms free with normal imaging.

## Conclusion

In conclusion this is the second case where carotid body tumour and associated thymoma has been reported. This case highlights the importance long term follow up of patients with HNPGLs that should be mandatory because of their association with other tumours.

Such cases should be seen in multidisciplinary clinic and should include geneticists as some of these cases are hereditary. Genetic tests should be offered to all of these patients so that proper surveillance by biochemical tests and imaging can be arranged and family members can be screened.
